# A novel and dual digestive symbiosis scales up the nutrition and immune system of the holobiont *Rimicaris exoculata*

**DOI:** 10.1186/s40168-022-01380-2

**Published:** 2022-11-05

**Authors:** Johanne Aubé, Marie-Anne Cambon-Bonavita, Lourdes Velo-Suárez, Valérie Cueff-Gauchard, Françoise Lesongeur, Marion Guéganton, Lucile Durand, Julie Reveillaud

**Affiliations:** 1 Univ Brest, CNRS, Ifremer, UMR6197 Biologie et Ecologie des Ecosystèmes marins Profonds, F-29280 Plouzané, France; 2 Univ Brest, INSERM, EFS, UMR 1078, GGB, F-29200 Brest, France and Centre Brestois d’Analyse du Microbiote (CBAM), Brest University Hospital, Brest, France; 3 MIVEGEC, Univ. Montpellier, INRAe, CNRS, IRD, Montpellier, France

**Keywords:** *Rimicaris exoculata*, Digestive symbiosis, *Hepatoplasmataceae*, *Deferribacteres*, Immunity, Metagenomics

## Abstract

**Background:**

In deep-sea hydrothermal vent areas, deprived of light, most animals rely on chemosynthetic symbionts for their nutrition. These symbionts may be located on their cuticle, inside modified organs, or in specialized cells. Nonetheless, many of these animals have an open and functional digestive tract. The vent shrimp *Rimicaris exoculata* is fueled mainly by its gill chamber symbionts, but also has a complete digestive system with symbionts. These are found in the shrimp foregut and midgut, but their roles remain unknown. We used genome-resolved metagenomics on separate foregut and midgut samples, taken from specimens living at three contrasted sites along the Mid-Atlantic Ridge (TAG, Rainbow, and Snake Pit) to reveal their genetic potential.

**Results:**

We reconstructed and studied 20 Metagenome-Assembled Genomes (MAGs), including novel lineages of *Hepatoplasmataceae* and *Deferribacteres*, abundant in the shrimp foregut and midgut, respectively. Although the former showed streamlined reduced genomes capable of using mostly broken-down complex molecules, *Deferribacteres* showed the ability to degrade complex polymers, synthesize vitamins, and encode numerous flagellar and chemotaxis genes for host-symbiont sensing. Both symbionts harbor a diverse set of immune system genes favoring holobiont defense. In addition, *Deferribacteres* were observed to particularly colonize the bacteria-free ectoperitrophic space, in direct contact with the host, elongating but not dividing despite possessing the complete genetic machinery necessary for this.

**Conclusion:**

Overall, these data suggest that these digestive symbionts have key communication and defense roles, which contribute to the overall fitness of the *Rimicaris* holobiont.

Video Abstract

**Supplementary Information:**

The online version contains supplementary material available at 10.1186/s40168-022-01380-2.

## Background

Microorganisms are involved in all geochemical cycles, growing as free-living organisms, organized communities, or undergoing symbiotic relationships. Host-associated microorganisms are essential in almost all animal lifestyles and life stages and are regarded as a driving force of evolution [1, 2]. As Angela Douglas wrote, *eukaryotes are not alone* [3]. Today, a host and its symbionts are even considered as a single entity, called a holobiont, sharing capabilities and expanding each other’s fitness [4]. In deep-sea hydrothermal environments where photosynthesis is not possible, studies have mainly focused on chemosynthetic symbioses fueled by reduced compounds emitted by vent fluids. Chemoautotrophic symbionts can be found in specialized organs/cells, such as in *Bathymodiolus* mussels and *Alviniconcha* gastropods, to cite just a few examples (for review see [5, 6]). Crustaceans also colonize extreme environments and harbor dense symbiotic communities on their cuticles [7–9]. In most of these examples, digestive organs are reduced but still present. However, little attention has been paid to their potential role and associated microbial communities.

*Rimicaris exoculata* is a hydrothermal vent shrimp that colonizes several active vent fields of the Mid-Atlantic Ridge (for review see [9]). It harbors a diversified symbiosis in its cephalothorax, fueling its host through transcuticular absorption of nutrients [10]. Although mainly symbiotrophic (up to 80% of carbon source) [11], it has a reduced but open digestive system, with a mouth, foregut (esophagus and stomach), hepatopancreas and midgut (part of the digestive tract), and hindgut (comprising rectum and anus) recently fully described [12]. In crustaceans, both the foregut and hindgut are of ectodermic origin and covered by a cuticle, lost at each exuviation. The midgut, of endodermic origin, has no cuticle and presents a brush border epithelium composed of basal cells with numerous microvilli [12]. In the midgut, a peritrophic membrane prevents any direct contact of ingested food with epithelial cells. This keeps the crustacean ectoperitrophic space free of microorganisms [13], but this is not the case for *Rimicaris* species where bacteria are observed inserted between epithelial cells microvilli [12, 14, 15].

In *R. exoculata*, the digestive tract is usually full of sulfurous minerals and some cuticle particles, but no other organic debris. It appears mostly empty after exuviation or after 72 h of starvation experiments [14], showing that the *Rimicaris* digestive tract is functional with active alimentary bolus transit. Moreover, a lengthy transit duration may enable nutrient absorption and microbial activity. A first study revealed carbon fixation in the *Rimicaris* digestive system [11]. A second study, based on 16S rDNA amplicon sequencing, focused on potential microbial colonization that may help shrimp nutrition or mineral detoxification. It revealed lineages related to cephalothorax ones (*Campylobacteria* and *Gammaproteobacteria*), but also two other lineages affiliated to *Mollicutes* (recently reclassified by GTDB as a *Bacilli* clade of *Firmicutes* [16], order *Mycoplasmatales*) and *Deferribacteres* [14].

*Mycoplasmatales* relatives associated with *Rimicaris* were separated into three lineages according to sampling location, one being restricted to the Rainbow site [15]. *Candidatus* Hepatoplasma crinochetorum, first identified in a terrestrial isopod hepatopancreas [17] was the most closely related bacteria. In this model, *Mycoplasmatales* are horizontally acquired by trophallaxis (i.e., a form of social feeding) from mother to offspring [18]. *Mycoplasmatales* relatives are thereby implied in host nutrition and immunity [18, 19]. In the deep marine scavenger isopod *Bathynomus* sp., living in a low food environment, the foregut harbors two *Mycoplasmatales* lineages, Bg1 and Bg2, implied in host nutrition and host protection against pathogens [20]. High-throughput 16S rDNA sequencing approaches did not retrieve *Mycoplasmatales* lineages in the environment surrounding *Rimicaris*. Yet, they were identified in both the midgut and foregut of the shrimp, with little genetic differentiation between both organs, possibly due to sample limitation [21]. These *Mycoplasmatales* lineages have also been identified in other shrimp hosts, such as the co-occurring deep-sea hydrothermal shrimp *Rimicaris chacei* [22]. This shrimp is both a symbiotroph and a scavenger, rather than strictly chemosymbiotrophic like its *R. exoculata* counterpart. However, *Mycoplasmatales* lineages have not been identified in reared shrimp [23] and the exact role of these lineages in *Rimicaris* shrimp remains to be investigated.

*Rimicaris*-associated *Deferribacteres* seemed affiliated to a single species-like group with ≥ 99% similarity based on 16S rDNA gene sequences, whatever the site, and were shown to be an active resident lineage using RT-PCR experiments [15]. However, differentiation appeared to occur, with broader bacterial diversification at the Rainbow site suggesting a more ancient and/or a larger holobiont population size at this location [15]. *Deferribacterales* relatives such as *Deferribacter abyssi* have been described in deep-sea hydrothermal vents like Rainbow, occurring as free-living anaerobic thermophilic bacteria, capable of iron reduction [24]. The *Deferribacteres* lineage identified in deep-sea shrimps such as *R. chacei* [22] or *R. exoculata* [14, 15] is most closely related to *Mucispirillum sp.* [25]. *Mucispirillum schaedleri* has been first isolated from the mucus layer of a laboratory rodent digestive system and can be grown in pure cultures, which is not the case for other symbionts [26]. This *Deferribacteres* lineage has not been recovered in the shrimp environment [21].

Abundant bacterial communities in the midgut of *Rimicaris* were observed by transmission microscopy [14]. Long filamentous single-cell bacteria are inserted between the microvilli of brush border midgut epithelial cells [14, 15], which is markedly different from what is known for most crustaceans [13]. These lineages, which are still present after long starvation experiments that empty the midgut (up to 72 h), appear to be residents. These long cells have a double membrane and are probably not affiliated to *Mycoplasmatales*, described as cell wall-less bacteria [27]. Low carbon fixation was also measured in the midgut [10, 11] and is possibly due to relatives of *Campylobacterota* and *Gammaproteobacteria* as in the cephalothorax [28], which possibly participate in host nutrition. However, to date, the role of this complex digestive microbiota and its control in the *R. exoculata* model remain unknown. Despite many attempts, no symbiont could be cultivated from the digestive organs of *Rimicaris*, greatly limiting our understanding of their roles.

To study the potential function and acquisition of the *Rimicaris* digestive microbiota, we used a metagenomic approach to investigate the digestive system of specimens from three hydrothermal vent locations, Rainbow, TAG, and Snake Pit, presenting contrasted geochemical environments [29]. We split the digestive system into two main parts, the foregut (including part of the esophagus) and the midgut. The hepatopancreas lysed during dissection was therefore discarded. Due to the small size of these organs in *Rimicaris* shrimps and the presence of high levels of inhibitors that hinder molecular approaches [14], we pooled nine to ten specimen organs per site. We then investigated the diversity, distribution, and potential role of the microbiome of each organ separately, including the use of fluorescence microscopy targeting long bacteria located in the ectoperitrophic space. We present the results of these investigations and discuss how the symbioses uncovered may complement each other, together with the well-studied one of the gill chamber.

## Methods

### Sample collection

The samples were taken from three hydrothermal sites on the Mid-Atlantic Ridge: Rainbow (36° 13′ N, 33° 54′ W, 2300-m depth) during the BioBaz cruise (August 2013, 10.17600/8010140), and both TAG (26° 08′ N, 44° 49′ W, 3660-m depth) and Snake Pit (23° 23′ N, 44° 58′ W, 3480-m depth) during the BICOSE2014 cruise (January 2014, 10.17600/14000100) and BICOSE2 cruise (February 2018 10.17600/18000004) for fluorescence microscopy samples (see Fig. 1 from [28] for a map of the sites). We collected *Rimicaris exoculata* specimens using a suction sampler manipulated by the Remotely Operated Vehicle “Victor 6000” or the Human Occupied Vehicle “Nautile,” operated from the research vessel “*Pourquoi pas?*”. After recovery, the samples were frozen directly on board at −80°C for later dissection of each organ and then genomic DNA extraction. For microscopy analyses, we dissected samples on board under sterile conditions to recover the midgut (M) and foregut (F), that we immediately fixed in 3% formalin for 3 h, rinsed with phosphate-buffered saline (1× PBS), and stored in PBS/ethanol at −20°C (1:1).

**Fig. 1:**
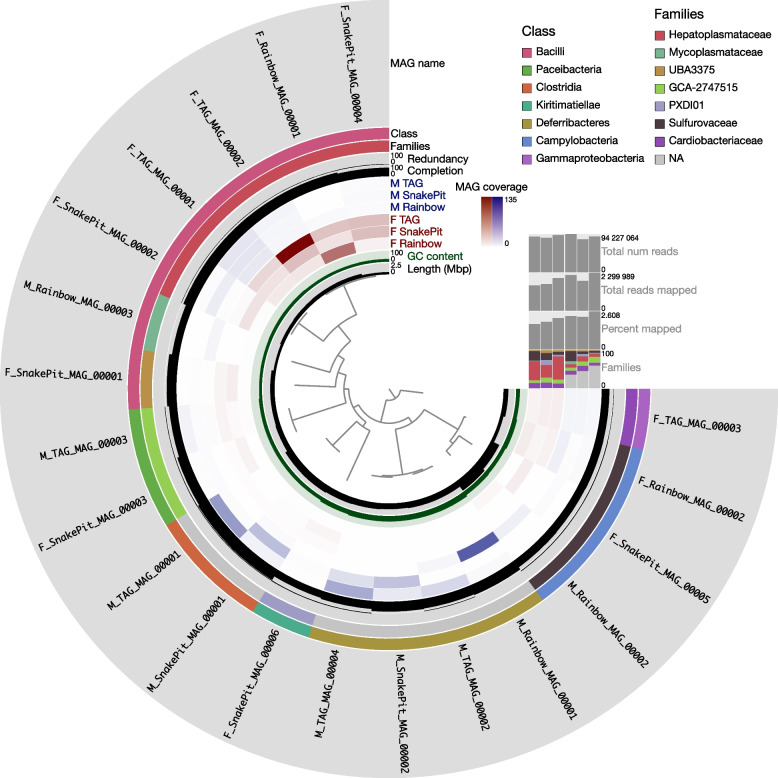
Static image from the anvi’o interactive display for the six *Rimicaris* midgut (M) and foregut (F) samples with the 20 metagenome-assembled genomes (MAGs) reconstructed in this study. From inner to outer layers: phylogenomic tree based on concatenated protein-coding genes according to GTDB-Tk, length genome layer, GC-content information about contigs stored in the contig database auxiliary layer, six view layers with mean coverage information about MAGs across samples stored in the profile database, percentage completion and redundancy, genome family and class based on GTDB-Tk, and MAG layer. The horizontal layers show the MAG taxonomy based on GTDB-Tk for families (with the relative abundance of families noted as percentages of reads recruited to the bins for each sample), percentage of reads mapped, total number of reads mapped, and total number of reads for each sample

### DNA extraction and sequencing

In the laboratory, we thawed and aseptically dissected on ice the digestive tract parts, foregut and midgut of ten specimens for Snake-Pit and TAG, and nine specimens for Rainbow. The tissues per organ and site were pooled, resulting in one foregut and one midgut sample for each of the three vent sites. We added 1 ml of TE-Na-1X lysis buffer (100 mM Tris-HCl; 100 mM NaCl; 0,5 mM EDTA, pH8) before filtration on 20-μm nylon filters (Millipore). We centrifuged the filtrates (9000 *g*, 15 min, 4°C) and treated the supernatant and pellet separately with the same method, although they were pooled afterward, as described below, before sequencing. For each fraction, we added 500 μl of 10% Sarkosyl, 500 μl of 10% SDS, and 100 μl of proteinase K (20 mg/ml) before incubation at 45°C for 3 h. We centrifuged digested samples for 5 min (6000 *g*) and extracted supernatants twice with cold phenol-chloroform-isoamyl alcohol (with a ratio of 24:24:1) and once with chloroform (mixed in a 1:1 ratio with lysates). We precipitated nucleic acids overnight with 2 volumes of −20°C absolute ethanol and centrifuged them for 5 min (11000 *g*). The latter were air-dried and suspended in 500 μl TE 1× (10 mM Tris-HCl, 2 mM EDTA, pH 7.5). The pooled nucleic acids obtained from pellets and supernatants were purified using an AMPure XP kit (Amersham Biotech). We performed metagenomic sequencing using an Illumina HiSeq3000 instrument with a paired-end read length of 2 × 150 bp with an Illumina TruSeq Nano kit on the GeT PlaGe platform (Castanet-Tolosan, France).

### Metagenomic analysis

We removed adapter sequences from the reads using bbduk from bbmap v38.57 (https://sourceforge.net/projects/bbmap/) and performed the following steps using the metagenomic snakemake workflow implemented in anvi’o v6 [30]. Briefly, we used illumina-utils v1.4 for sequence quality filtering [31]. We assembled individual metagenomes using Megahit v1.2.9 [32] with the meta-sensitive mode and a minimum contig length of 1000 bp. We identified open reading frames using Prodigal v2.6.3 [33] and single-copy core genes using HMMER v3.2.1 [34] along with a collection of built-in HMM profiles for bacteria and archaea. We annotated gene functions using the NCBI Clusters of Orthologous Groups (COG) [35] and the KOfam HMM database of KEGG orthologs [36, 37]. We recruited metagenomic short reads to contigs using Bowtie2 v2.4.2 [38] and converted SAM to BAM files using samtools v1.7 [39]. We profiled the BAM files using “anvi-profile” and combined all profiles into a single anvi'o profile with the program “anvi-merge.” We used CONCOCT v1.1.0 [40] to group contigs into bins and manually refined them with the “anvi-refine” program. We only kept Metagenome-Assembled Genomes (MAGs) with completion above 60% and contamination under 10% for the next steps. We named final MAGs according to the following scheme: name of the MAG including the prefix “M” or “F” to specify the type of sample used for the assembly of the MAG (for midgut or foregut respectively), followed by the sampling site (TAG, Snake Pit, or Rainbow), the “MAG” abbreviation and a number, where for each assembly the MAGs had a number that starts with “00001” and increments to the maximum number of MAGs that were retained from that assembly. As independent assembly of metagenomes from similar environments can reconstruct almost identical genomes, we dereplicated our MAGs collection. We selected a unique representative genome for MAGs showing more than 99% average nucleotide identity (ANI) with a coverage threshold of 50% using dRep v2.3.2 [41]. We then performed a final mapping of all metagenomes on the dereplicated MAGs to calculate their mean coverage and detection.

To estimate the relative abundance of symbionts, we retrieved the raw counts mapping to each MAG using samtools view [39]. We normalized data within and between samples with the gene length corrected trimmed mean of M-values (GeTMM, [42]), using genome length instead of gene length). We used DESeq2 [43] to analyze differences in MAG abundance between organs. Differentially abundant MAGs with adjusted *p* values of 0.01 (padj < 0.01) and absolute log2-fold changes of 1.5 were considered significant in this study.

We made the taxonomic affiliation of our MAGs with the classify workflow of GTDB-Tk v1.5.0 [44] and created a multiple sequence alignment based on the 120 amino acid marker set of our *Deferribacteres* and *Hepatoplasmataecae* MAGs and their closely related taxa using the GTDB-Tk “align” command. We trimmed multiple sequence alignments using TrimAl v1.4.1 [45] by removing positions with gaps in more than 50% of sequences. We reconstructed a maximum likelihood phylogenetic tree using IQ-TREE v2.0.3 [46] with the “WAG” general matrix model and 1000 bootstrap replicates that we visualized using FigTree v1.4.4 (https://github.com/rambaut/figtree). We calculated pairwise genomic average nucleotide identity gANI using “anvi-compute-genome-similarity” from anvi’o with the pyANI program. We extracted KO assignments from the anvi’o database and used KEGG Decoder v1.2.2 [47] to estimate the completeness of various metabolic pathways. We used dbCAN2 v2.0.6 [48] to predict the genes encoding carbohydrate-active enzymes (CAZymes), which combines three databases/annotation methods for CAZymes. We kept CAZymes annotations found by at least two annotation methods as suggested by [48]. We investigated the presence of clustered regularly interspaced short palindromic repeats (CRISPR)-associated (Cas) system using CRISPRCasFinder release 4.2.20 [49], selecting MAG contigs with CRISPR showing an evidence level of 4. Schematic representations of predicted metabolic potentials for both *Deferribacteres* and *Hepatoplasmataceae* MAGs were constructed based on the KEGG annotations and KEGG Mapper/Reconstruct tool (https://www.genome.jp/kegg/mapper/reconstruct.html). If a gene was missing from a pathway, it was investigated through COG annotations. To examine the taxonomic composition of each sample based on the small-subunit rRNA, we analyzed the quality-filtered reads using the phyloFlash v3.4 pipeline [50] with the option “almost everything” and the SILVA database release 138.1 [51].

### Code and data availability

The raw reads of the metagenomes are available in the European Nucleotide Archive under Bioproject Accession Number PRJEB50056. The URL https://gitlab.ifremer.fr/rimicaris/rimicaris-exoculata-gut-epibionts-metagenomes provides access to a detailed reproducible bioinformatics workflow for all the computational analyses. We also made the following files available online: FASTA files for individual metagenomic assemblies, FASTA files for the 21 MAGs, and anvi’o merged profile databases of the 20 dereplicated MAGs (10.12770/437f34c4-aba3-4962-9f6f-4c469defc483).

### Procedures for fluorescence microscopy

Fixed samples were treated in the lab as described in [12, 14]. Briefly, we embedded dehydrated samples in polyethylene glycol distearate-1-hexadecanol (9/1) resin (Sigma, St. Louis, MO), and blocks were cut into 8-μm sections using an RM 2255 microtome (Leica, Wetzlar, Germany). Resin was removed before using the sections.

In order to locate and identify the ectoperitrophic space symbionts, sections were treated for fluorescence in situ hybridizations (FISH). We hybridized them in 30-μl hybridization buffer containing 30% formamide with 2 μl of a newly designed 8-μM probe Def1229-Cy3 (5′-GCCCTCTGTATAGTCCATTG-3′) specific to the *Deferribacteres* symbiotic lineage, for 3 hours at 46°C and washed sections at 48°C for 15 min [12]. Sections were mounted on slides with SlowFade™ Gold antifade reagent containing 40-6-diamidino-2-phenylindole (DAPI) (Invitrogen). We performed our observations using an Imager.Z2 microscope equipped with the ApoTome.2 sliding module and Colibri.7 light technology (Zeiss, Oberkochen, Germany). Micrographs were made with Zen software (Zeiss).

In order to observe bacterial chromosomes and count their number per cell in the midgut symbionts, labeling was realized with the YOYO™-1dye (Invitrogen), a highly DNA-specific dye. Sections were then labeled in a 30-μl aqueous solution containing 10^-4^ mM YOYO™-1dye for 15 min at room temperature and then washed with water. Sections were mounted on slides with Citifluor AF1 antifade solution (Electron Microscopy Sciences, Hatfield, PA, USA). We performed observations using a LSM 780 confocal microscope (Zeiss) using the photon counting method to reveal the fluorescence and analyze/quantify it as a spectrum. Micrographs were done with Zen software (Zeiss).

## Results and discussion

### The reconstruction of 20 *Rimicaris*-associated digestive tract Metagenome-Assembled Genomes (MAGs)

Our study focuses on three foregut (F) and midgut (M) metagenomes from three contrasting hydrothermal sites along the Mid-Atlantic Ridge: Rainbow, TAG, and Snake Pit showing different geochemical signatures [52]. Briefly, fluids are more enriched in methane, hydrogen, and iron at Rainbow (an ultramafic site) and sulfur at TAG and Snake Pit (two basaltic sites), which can drive dedicated microbial chemoautotrophic activities [28]. Of note, contrasted sites allowed a better resolution for differential coverage and MAG reconstruction. However, we did not have enough individual organ samples from each site to investigate the differences between sites. We reconstructed the MAGs of each site to examine the functional potential of the *Rimicaris* digestive tract microbiome at the genome-resolved scale. Shotgun sequencing of total community DNA recovered from the *Rimicaris* digestive tracts resulted in 677 million reads with an average of 113 million reads per metagenome (Supplementary Table S1). Assembly of each sample resulted in 103K to 147K contigs longer than 1 kbp, which recruited on average 57.60% of high-quality reads. Our automatic and manual binning combination allowed us to reconstruct 21 MAGs with a 60% minimum completion and a 10% maximum redundancy. Dereplication eliminated one MAG M_SnakePit_MAG_0003 (*Hepatoplasmataceae*), giving a final collection of 20 MAGs. Metagenome-Assembled Genome statistics on genome size, number of contigs, N50, GC%, percent of completion and redundancy, and taxonomic affiliation are detailed in Supplementary Table S2.

Read recruitment revealed that between 1.75 and 2.61% of the high-quality reads mapped to the final MAG collection (Supplementary Table S1). This low percentage of recruitment can be explained by host DNA contamination [53], which was confirmed with a small unit rDNA taxonomic investigation using phyloFlash (see Supplementary Figure S1). MAG Taxonomic affiliation inferred from single-copy core gene analyses (Fig. 1, Supplementary Table S2) showed the presence of *Bacilli* (7 MAGs), *Deferribacteres* (4 MAGs), *Campylobacteria* (3 MAGs), *Clostridia* (2 MAGs), *Paceibacteria* (2 MAGs), *Kiritimatiellae* (1 MAG), and *Gammaproteobacteria* (1 MAG) classes.

These results were in agreement with the predominant lineages recovered using 16S rDNA genes in phyloFlash (Supplementary Figure S1). It should be noted that recovered *Paceibacteria*, *Kiritimatiellae*, *Campylobacteria*, and *Gammaproteobacteria* classes were also found in the cephalothorax, but these MAGs belong to distinct lineages and were present in low abundance [28]. The remaining *Bacilli*, *Deferribacteres*, and *Clostridia* MAGs appeared as specific to the *Rimicaris* digestive tracts. An overview of the functions of the MAG community and the metabolic pathways using the KEGG decoder is given in Supplementary Figure S2.

### Digestive tract and organ-specific dominant lineages

**Fig. 2:**
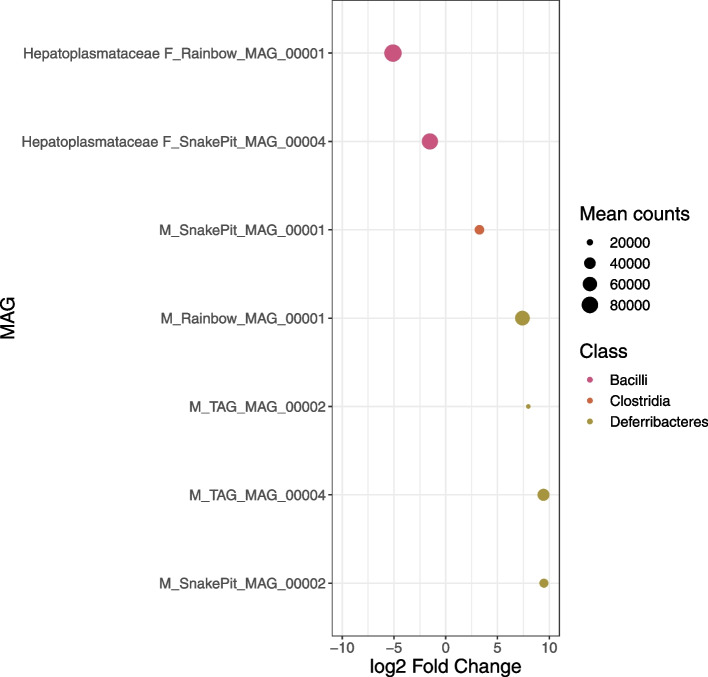
Differentially abundant MAGs between organs identified through DESeq2 of midgut vs. foregut. Each line on the *y*-axis indicates a MAG. MAG family is indicated on the left of the MAG name when they are assigned until this level. MAGs dots are colored according to the class level and size of the dots corresponds to mean read counts after normalization with GeTMM and DESeq2 (corresponding to baseMean). Positive and negative log2FoldChange values indicate differentially abundant MAGs in midguts and foreguts, respectively. Cutoff values for visualization in the plot were 0.01 for padj and 1.5 for log2FoldChange absolute value

Overall, we observed dominant and organ-specific symbionts, but with distinct lineages belonging to the same family at each sampling site (Fig. 1, Supplementary Table S2). The *Hepatoplasmataceae* family (*Bacilli* class, *Mycoplasmatales* order) and the *Deferribacteres* class seemed to dominate the foregut and midgut, respectively. A DESeq2 analysis confirmed that two MAGs affiliated to the *Hepatoplasmataceae* family (F_Rainbow_MAG_0001 and F_SnakePit_MAG_0004) were significantly more abundant in the *Rimicaris* foregut than in the midgut (log2FoldChange < -1.5, Fig. 2).

On the contrary, we observed the four *Deferribacteres* MAGs (M_Rainbow_MAG_00001, M_TAG_MAG_00002, M_TAG_MAG_00004, and M_SnakePit_MAG_00002) in much higher abundance in midguts than in foreguts (log2FoldChange > 1.5). MAG M_SnakePit_MAG_0001 affiliated to the *Clostridia* class was also more abundant in the midgut. We analyzed the dominant foregut and midgut lineages (Fig. 1, Supplementary Table S2), composed of MAGs from the *Hepatoplasmataceae* family and MAGs from the *Deferribacteres* class, more thoroughly.

### *Rimicaris* foregut harbors novel *Hepatoplasmataceae* lineages with highly reduced and symbiotic genome characteristics

**Fig. 3:**
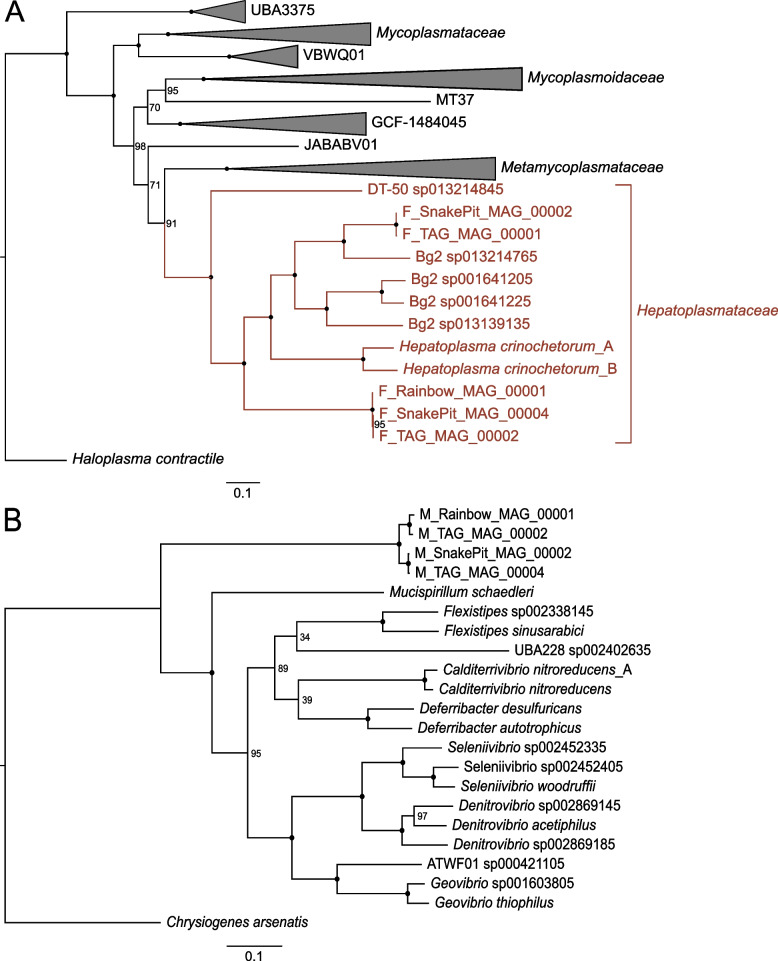
Maximum likelihood phylogenetic tree using IQ-TREE v2.0.3 with the “WAG” general matrix model and 1000 bootstrap replicates visualized using FigTree for **A** *Hepatoplasmataceae* and **B** *Deferribacteres* MAGs and their closest relatives. Nodes represented by a dot indicate a bootstrap value of 100; lower values are specified. *Ca.* Hepatoplasma crinochetorum Ps and *Ca.* Hepatoplasma crinochetorum Av are noted A and B, respectively, in GTDB-Tk

Out of seven *Bacilli* MAGs reconstructed from the foregut, five were affiliated to the *Hepatoplasmataceae* family (Fig. 3A).

Precisely, our phylogenomic comparison showed two clusters: one composed of two MAGs affiliated to the GTDB Bg2 genus identified in a deep-sea isopod scavenger [20], both sharing 97.6 to 98.2% ANI, and another one comprising three MAGs sharing 95.7 to 98.3% ANI between each other with no genus classification, but with *Candidatus* Hepatoplasma crinochetorum as closest relative through GTDB-Tk (Fig. 3A and Supplementary Table S2). *Candidatus* Hepatoplasma crinochetorum was first identified in terrestrial isopods *Porcellio scaber* [17] and *Armadillium vulgare* [54] where they colonize the hepatopancreas, the main digestive and adsorption organ in terrestrial isopods [19]. These isopods feed on degrading litter and wood, leading to an imbalanced diet [17]. Their digestive symbionts have been suggested to be involved in the hydrolysis of complex organic matter (leaves litter), releasing nutriments for themselves and their host [19]. To our knowledge, all attempts to cultivate *Mycoplasmatales* relatives have failed [20], but these lineages may share commonalities in these two organs, i.e., the hepatopancreas in terrestrial hosts and the midgut in deep-sea ones.

**Fig. 4:**
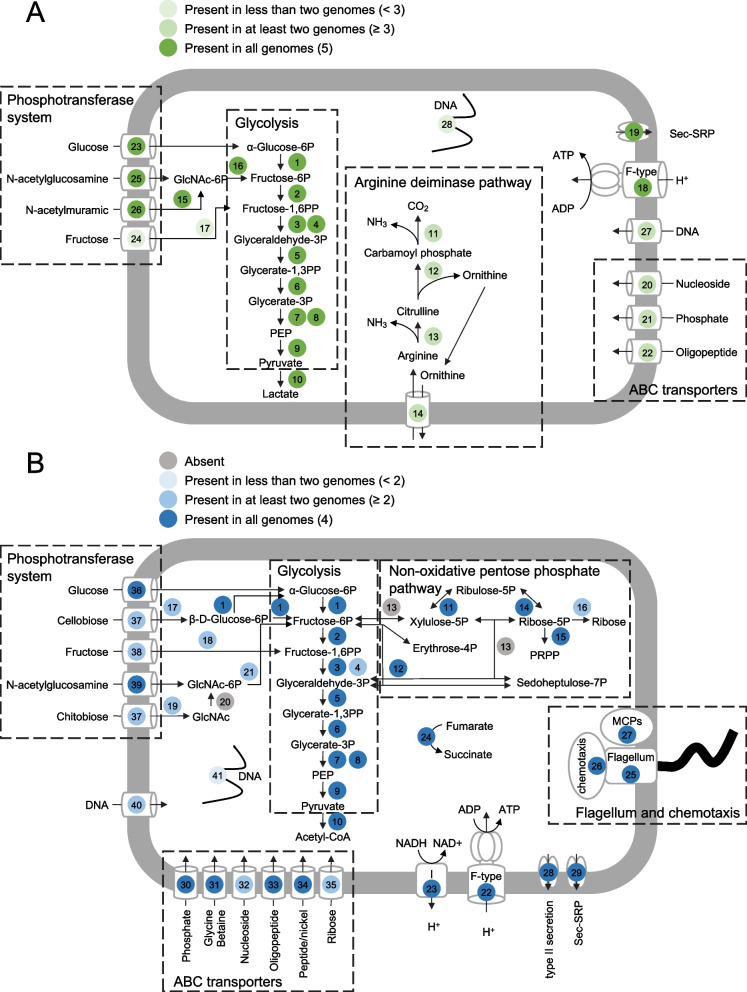
Schematic representation of the predicted metabolic potentials within **A** the 5 *Hepatoplasmataceae* and **B** the 4 *Deferribacteres* MAGs using the KEGG annotations and the KEGG Mapper/Reconstruct tool. Genes present in all, at least half, or less than half are indicated in differentially contrasted colors. The metabolic potentials are focused on carbon metabolism, transporters, flagellum, and chemotaxis. Full names and gene copy numbers are detailed in Supplementary Table 4

*Hepatoplasmateceae* MAGs of *Rimicaris* all showed typical characteristics known from other *Mycoplasmatales* genomes [54], including a low G+C content (from 22.42 to 26.56%), a highly reduced genome size (from 0.48 to 0.83 Mbp) (Supplementary Table S2), and a truncated metabolic pathway. Interestingly, the *Hepatoplasmatacea* MAGs reconstructed herein also lacked a set of eight Single Copy core Genes (SCG), emphasizing the exceptionally streamlined and reduced symbiotic genomes (Supplementary Table S3). In addition, as for its closely related *Candidatus* Hepatoplasma crinochetorum genomes [54, 55], genes for the tricarboxylic acid cycle (TCA), the pentose phosphate pathway, and the respiratory chain, except an F-type ATPase, are missing in all the five MAGs (Fig. 4A, Supplementary Table S4, and Supplementary Figure S2).

### Energy and carbon metabolism

All genes for glycolysis were found in the five *Hepatoplasmataceae* MAGs, implying that these lineages may use it to produce energy. Because its end-product is lactate, it may lead to cell acidification. Three protein-coding genes involved in the arginine deiminase pathway (ADI, ornithine carbamoyl transferase, and carbamate kinase) were found in the three *Hepatoplasmataceae* MAGs (Fig. 4A). The ADI pathway is found in several bacteria, including lactic acid bacteria. This process generates energy through ATP production and ammonia, leading to environment alkalization [56] and helping the survival of bacteria during acid stress. A putative transporter, YfcC (COG1288), is encoded in the *arcAFC* cluster of the three latter MAGs, with genes showing 75.71 to 76.84% identity with the arginine/ornithine antiporter of *Candidatus* Hepatoplasma crinochetorum. The YfcC transporter allows the cell to eliminate the ornithine end-product while importing a new arginine substrate [57].

We did not find any gene encoding CAZYmes within our *Hepatoplasmataceae* MAGs using dbCAN2 (Supplementary Figure S3). Thus, MAGs do not appear to have the ability to hydrolyze complex polysaccharides such as chitin. However, three MAGs possess the genes for *N*-acetylglucosamine transport and hydrolysis, an intermediate of chitin degradation that can be used in glycolysis after hydrolysis. So, *Hepatoplasmataceae* MAGs could be a secondary user of complex molecules that have already been broken down, possibly complementary to host chitinase [58]. Although most of the *Rimicaris* diet is fueled by autotrophic activity of the gill chamber symbiont [10, 28], pieces of cuticle are often observed in the alimentary bolus [14, 59]. The current data support the hypothesis that a heterotrophic source of refractory organic matter is essential for the shrimp, possibly after molts, to help with synthesis of new cuticle. At the end of the molt cycle, gill chamber symbionts appear lysed and are lost during exuvia [9], therefore, not contributing to shrimp nutrition. This hypothesis is consistent with observations made on terrestrial and deep-sea isopods, where intestinal microbes contribute to leaf-based nutrition of the hosts [19, 20]. This is also observed for another emblematic deep-sea vent chemosymbiotic holobiont, *Alviniconcha marsindica*, which harbors digestive symbionts that complement host nutrition primarily fueled by its gill symbionts [53].

### Amino acid and vitamin synthesis, transporters, and mobility

Pathways for nucleotide synthesis and vitamin or amino acid synthesis are missing or incomplete in these MAGs (Supplementary Figure S2) and suggest symbionts could rely on their host or other symbionts for these molecules. Only glycine production via serine and/or vice versa seems possible, through the glycine hydroxymethyltransferase gene. However, the two *Hepatoplasmataceae* MAGs affiliated to the GTDB Bg2 genus have genes coding for ABC-type dipeptide/oligopeptide transporters that may supply small peptides, which can then be degraded into amino acids (oligopeptidase, proteases) to complement cell requirements. Genes for phosphate transporters, nucleosides, and DNA, such as the ComEC competence protein, were also present in several MAGs. Degradation of DNA molecules may supply cells with phosphate, ribose, and bases, ensuring DNA replication in these bacteria. *Hepatoplasmataceae* MAGs lack flagellar or chemotaxis genes like their close relatives [17].

### Defense systems

CRISPRs/Cas systems were investigated in the MAGs (Supplementary Table S5) as they have been identified to provide acquired immunity against viruses and plasmids in bacterial and archaeal genomes [60]. MAG F_Rainbow_MAG_00001 showed a contig, F_Rainbow_MAG_00001_000000000038, that carries a CRISPR/Cas class 2 type II with 3 *cas* genes (*cas1/cas2/cas9*) together with repeated sequences of 36 bp and 46 spacers. The most similar homolog of *cas9* gene was from *Candidatus* Hepatoplasma crinochetorum from the terrestrial isopod *Armadillidium vulgare* [54] (33.6% identity at amino acid level). CRISPRs were also observed in MAG F_SnakePit_MAG_0004 and F_TAG_MAG_0002, but no *cas* genes were associated with the respective contigs. Despite their small genome size and their parasitic lifestyle, *Mycoplasmatales* are currently the bacteria with the smallest genome in which CRISPR/Cas systems have been reported [61] while these immune systems are typically absent in obligate symbionts [62]. Gut symbionts, as *Mycoplasmatales*, can be more exposed to phage attacks as compared to intracellular symbionts, which may explain the presence of phage defenses in these genomes [63]. The CRISPR/Cas system, if efficient, may be involved in the defense of the bacteria. It could also benefit the host by preventing dysbiosis resulting from phage attacks. Host defense by CRISPR/Cas systems of an holoturian’ s endosymbiont has been previously suggested [64]. Defense against foreign DNA in MAG F_SnakePit_MAG_0002 could also be achieved using the restriction-modification system *hsdMSR*, a system that has also been observed in *Ca.* Hepatoplasma crinochetorum Ps [55].

### The *Rimicaris* midgut contains a novel symbiotic Deferribacteres family

The four *Deferribacteres* MAGs reconstructed from midgut samples were closely related (Fig. 3B), but shared only 87.0 to 97.6% ANI, possibly suggesting that there are two different species (Supplementary Table S6). MAG affiliation through GTDB-Tk could not be done beyond the class level, suggesting a new family. *Deferribacteres* MAGs showed a relatively high G+C content (from 46.67 to 47.6%) and reduced genome size (from 1.25 to 1.36 Mbp, Supplementary Table S2) compared with the reference genomes of *Deferribacter desulfuricans* (38% GC and 2.2 Mbp together with a megaplasmid of 0.3 Mbp [65]), *Denitrovibrio* sp. (42.5% GC and 3.3 Mbp [66]), and *Mucispirillum schaedleri* (31% GC and 2.3 Mbp [25]). *Deferribacteres* have been identified in the gut of *R. exoculata* using 16S rDNA gene analysis, and the closest relative was the *M. schaedleri* strain UNSW I23 [15], which is confirmed here (Fig. 3B). It is an anaerobic but oxygen-tolerant, Gram-negative, and appears as spiral-shaped rods with bipolar flagella for motility. The sequencing of two *M. schaedleri* genome variants indicated intimate interactions with its host and many horizontal gene transfers with other symbiotic lineages, mostly *Campylobacterota* and Firmicutes [25].

### Energy and carbon metabolism

Like *Mucispirillum*, their closest relatives*,* the four reconstructed *Deferribacteres* MAGs in this study harbor genes for the complete glycolysis pathway (Embden-Meyerhof-Parnas EMP) and a nonoxidative pentose phosphate pathway (although the transaldolase E.C. 2.2.1.2 could not be identified in the four genomes, which is consistent with *M. schaedleri* [25]) (Fig. 4B, Supplementary Table S4).

Chitin in hydrothermal vent environments is an abundant source of carbon as they are believed to be among the highest chitin-producing systems [67]. Genes coding for CAZYme glycoside hydrolase family 19 were identified in three *Deferribacteres* MAGs using dbCAN2, suggesting they could be able to degrade chitin (Supplementary Figure S3). *Rimicaris* have a very short molt cycle duration, 10 days, and so must synthetize de novo its cuticle [68], being a huge cost of energy and nutriments. Chitin is a large and complex polymer, first degraded to chitobiose, a dimer of *N*-acetyl-d-glucosamine (GlcNac), which can then enter the bacterial cell through transporters or in monomers that are then imported into the cell (i.e., using *nagE* PTS transporters, whose coding genes were also retrieved in all our *Deferribacteres* MAGs). Most of the MAGs showed genes coding for PTS transporters for cellobiose, glucose, and fructose transport together with the genes necessary for their hydrolysis (*celF*, *fruK*) for use before glycolysis pathways. It is noteworthy that cellobiose transporters can also be involved in chitobiose transport. Genes involved in chitobiose degradation were almost all present, although the *N*-acetylglucosamine kinase (*nagK*) gene was not found in the four MAGs reconstructed in this study. The latter enzyme is required for GlcNac phosphorylation before entering the glycolysis pathway. Nevertheless, chitobiose can still be degraded in the periplasmic space or even in the outer medium and then GlcNac may be phosphorylated during import using the PTS transporter. GlcNac is also a bacterial cell wall component, contributing to peptidoglycan composition together with *N*-acetylmuramic acid (MurNAc). Chitin recycling may, therefore, help the host with chitin turn over and be used for symbiont cell wall synthesis.

None of the four *Deferribacteres* MAGs contained genes for a complete TCA cycle nor seemed to have those necessary to reduce nitrates (Supplementary Figure S2). We, nevertheless, detected genes coding for fumarate reductase as for the *M. schaedleri,* suggesting that fumarate may serve as a terminal electron acceptor (instead of oxygen under anaerobic conditions, Supplementary Table S4). Interestingly, we detected 11 of the 13 different subunits encoded by the so-called *nuo* genes that make the proton-pumping NADH: ubiquinone oxidoreductase respiratory complex I in *Escherichia coli* (Supplementary Table S4). The latter couples the transfer of electrons from NADH to ubiquinone with the translocation of protons across the membrane. The *nuo* genes were absent from the closely related genomes of *M. schaedleri.* However*,* they were present in some more divergent and free-living *Deferribacteres* like *Deferribacter desulfururicans* [65] or *Denitrovibrio acetiphilus* [66].

### Amino acid and vitamin synthesis, transporters

The only complete amino acid biosynthesis pathways included serine, glycine, and l-alanine (Supplementary Figure S2). l-alanine can be obtained from pyruvate using a NAD(H)-dependent l-alanine dehydrogenase (EC 1.4.1.1) [69]. Other amino acid biosynthesis pathways were incomplete or absent. These data suggest that *Deferribacteres* symbionts might depend on the host (or other symbionts in the gut or gill chamber) for amino acid supply. The four *Deferribacteres* MAGs nevertheless encoded genes for multiple ABC transporters for phosphate, nucleoside, and oligopeptide, which may supply the cell for amino acid and DNA synthesis.

In addition, the four MAGs contained the genes required for biotin and riboflavin biosynthesis, suggesting a vitamin synthesis role for these *Rimicaris*-associated bacteria. These gut microbes may provide supplementary vitamins for their *Rimicaris* hosts, although knowledge on the actual host metabolism remains limited due to the lack of a *Rimicaris* reference genome. Notably, the four MAGs did not show the pathway for de novo biosynthesis of coenzyme B12 (cobalamin coenzyme) reported in their close relative *M. schaedleri.*

### Mobility and secretion systems

The four genomes showed more than 30 genes required for flagellum biosynthesis and several chemotaxis-related protein-encoding genes that were also found in the *M. schaedleri* variants MCS and AYGZ (Fig. 4B and Supplementary Table S4). The presence of flagella is involved in host-symbiont recognition and colonization as it enables symbionts to move toward their host before infestation. Chemotaxis genes (chemoreceptors) are involved in the detection of host molecules, and some are also implied in the functioning of flagella (genes *che* or *tlp* [70]). They also contained genes for a type II secretion system, which symbionts may use to colonize hosts, together with a Sec-SRP, like its closest relatives, although they did not show any type VI nor Tat systems. In hydrothermal vent ecosystems, even sensitive methods have failed to detect bacterial digestive lineages in shrimp environments [21], although *Rimicaris* cephalothorax symbionts have been retrieved [21, 71]. Along *Rimicaris* juvenile metamorphosis toward the adult stage, these symbiont lineages may colonize the midgut, thanks to these genes encoding flagella.

### Defense systems

Immunity systems were observed in two *Deferribacteres* MAGs (M_Rainbow_MAG_00001 and M_TAG_MAG_0004) containing CRISPRs with 30 and 19 spacers, respectively, but both without the *cas* gene (Supplementary Table S5). Both CRISPR arrays were located at the end of the contig. The *cas* gene could have been lost during assembly, as repetitive genome regions are often refractory to assembly using short-read sequencing technologies. The localization of the *Deferribacteres* within the microvilli as described below could also protect the symbionts from phage attack and explain the incomplete CRISPR/Cas systems in these MAGs.

#### Location and cell morphology of Deferribacteres

**Fig. 5:**
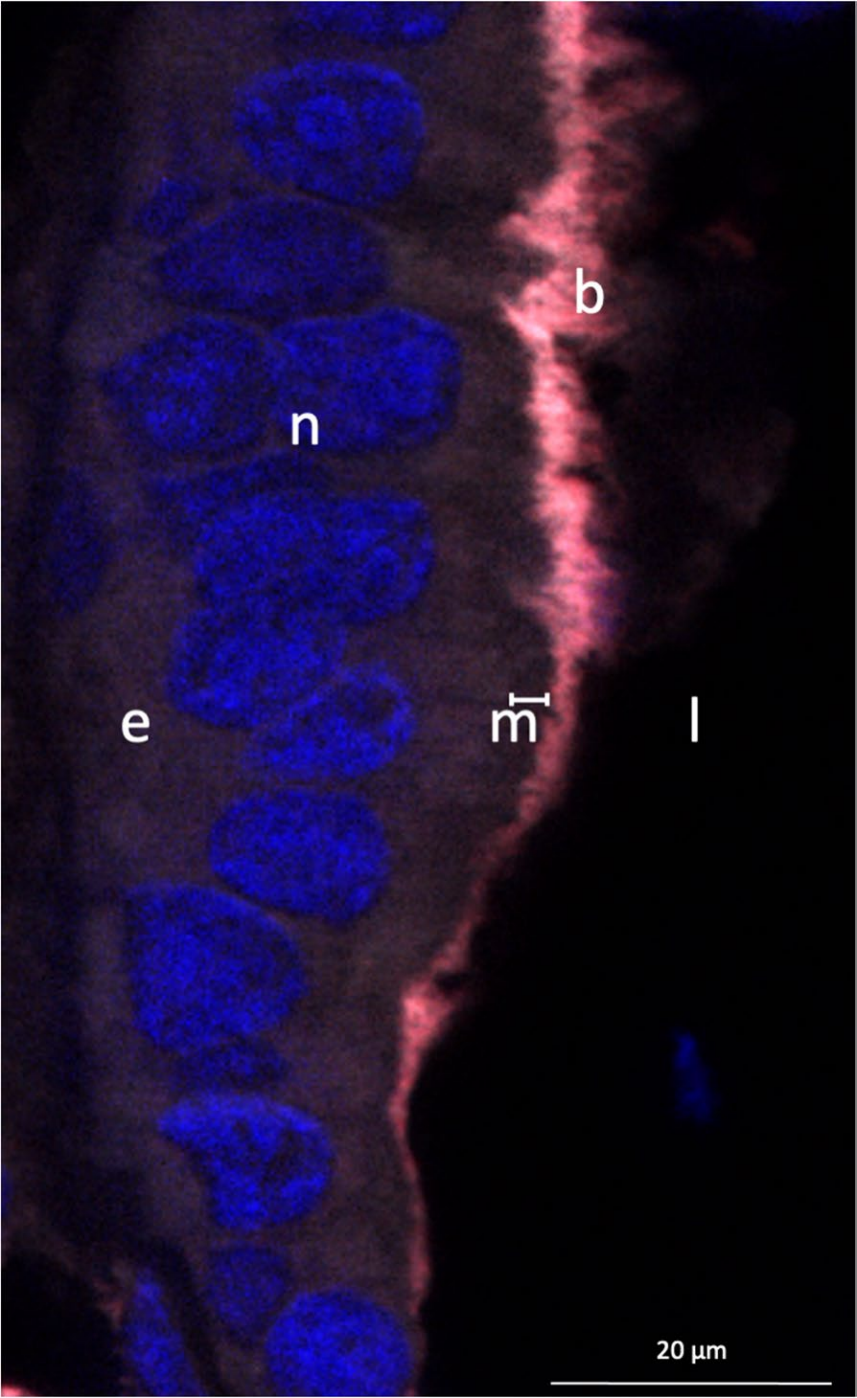
FISH observation of *Deferribacteres* cells in the midgut of an adult male *R. exoculata* using newly designed probes [12]. These long filamentous bacteria (b) are inserted between the microvilli (m) of the intestine epithelium (e) of the host. Eukaryote nuclei (n) at the center of epithelial cells are labeled with DAPI (blue). The apical brush border of the epithelium with the microvilli (represented by the white line just beside the legend m) is all along the lumen (l). Bacterial cells (b) are co-hybridized with the Eubacterial probe (Eub338, colored in white on the image) and with the specific *Deferribacteres* symbiont probe (Def1229, colored in red on the image), so they are visible in pink on the image

In previous studies, long single-cell filamentous bacteria were observed in the ectoperitrophic space, inserted between the microvilli of the digestive tract basal cells [14, 15]. These bacteria have here been identified using fluorescent in situ hybridization microscopy with newly developed specific probes as belonging to the *Deferribacteres* class (Fig. 5) [12].

**Fig. 6:**
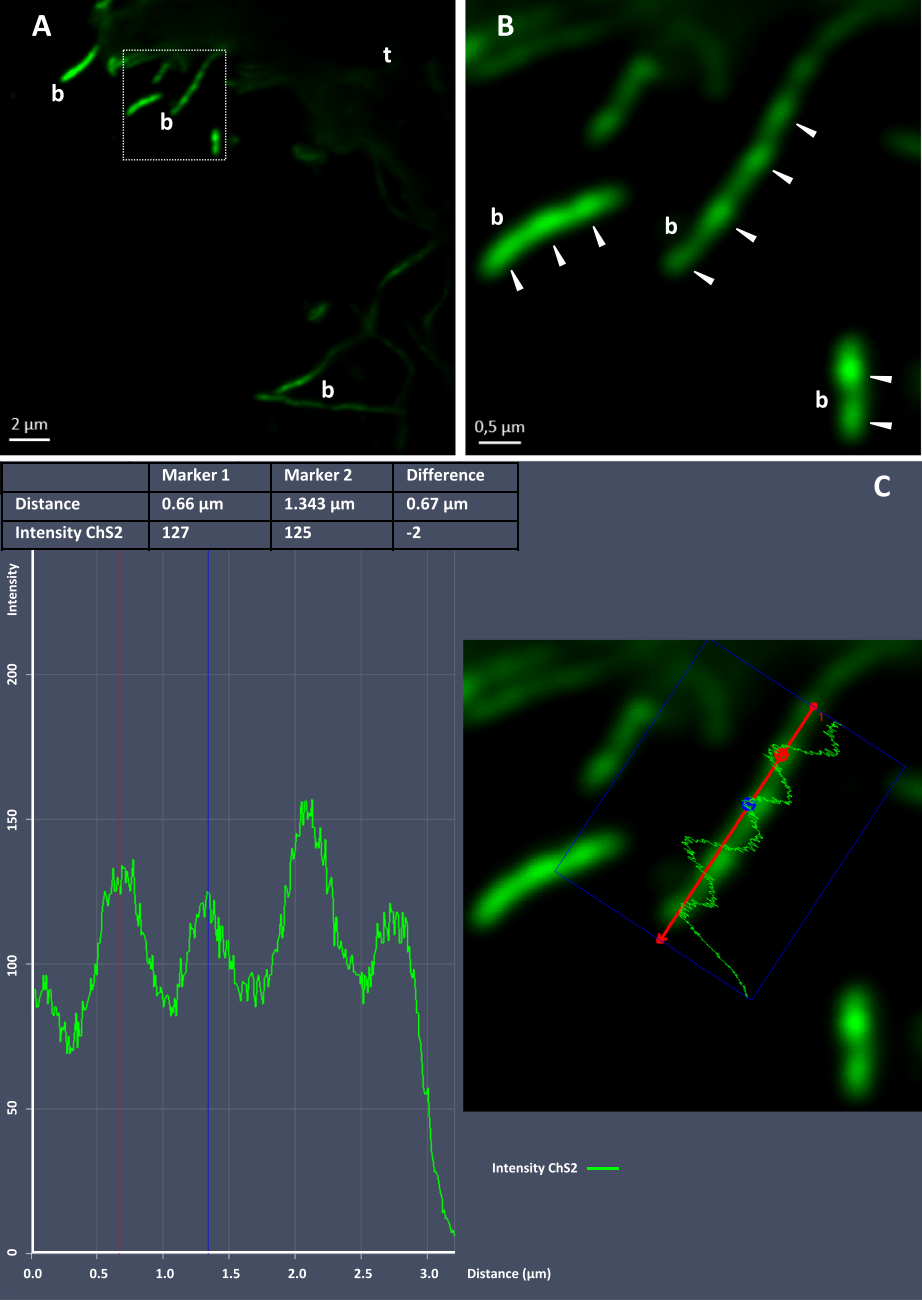
Chromosome observation of the long filamentous bacteria using YOYO™-1 labeling. DNA (in green) was specifically labeled using the YOYO™-1 dye. The host nucleus is not visible here as being at the opposite of the microvilli of the epithelial host tissue (t). **A** Long filamentous bacteria (b) along the midgut brush-border cells. The white square is image **B** with a closer view. The scale bar represents 2 μm. **B** YOYO™-1 labeling reveals numerous bacterial chromosomes per cell (green balls). Cells are non-segmented and white arrows point out each chromosome. The scale bar represents 0.5 μm. **C** Fluorescence intensity profile within a bacterial cell revealing that chromosomes are regularly spaced by 0.6 to 0.7 μm

We can propose a hypothesis for this long single-cell morphology: a strong host control avoiding infestation of host cells after colonization in the ectoperitrophic space, a space usually described as sterile in most crustacean [13]. Here, all four *Deferribacteres* MAGs contain genes for complete cell division machinery and chromosome replication like their closest relative *M. schaedleri* (Supplementary Table S4), showing a copy of each gene for chromosome duplication (S phase) before Z-ring formation (*ftsZ* and relatives). According to our results using YOYO™-1 labeling (Fig. 6), cells do not undergo division ([14] and Fig. 5), but the chromosomes replicate, appearing in several copies per bacterial cell.

So, the host probably prevents bacterial cell division but not chromosome replication, leading to morphologies similar to those observed in bacterial cultures supplemented with division inhibitors [72, 73]. The host may control this division using antimicrobial peptides (AMP) such as Re-crustine. This AMP is implied in symbiont (*Campylobacteria* and *Gammaproteobacteria* at least) selection and attraction, as symbionts were observed embedded with AMP without any lysis form in the cephalothorax [74]. Nevertheless, Re-crustine may have other effects on other bacterial lineages. As discussed by Le Bloa et al. [74], this AMP is also probably only part of the host cocktail to control symbiosis. *Deferribaceteres* MAGs revealed several genes implied in stress detection, such as *groEL* and *hsp70*. These genes are known to be expressed under stressful conditions, in changing environments (temperature, chemicals), or during sensing of host control molecules such as toxins or AMP, for example. In mosquitoes, *the Asaia* symbiont enhances AMP synthesis by its host and contributes to its own selection as it is not the target of the antimicrobial peptide directed toward other microbes [75]. In both arthropod examples (shrimps and mosquitoes), symbionts may enhance host defense against pathogens or cheaters, favoring their own selection and colonization. All these observations open perspectives regarding the universality of symbioses in terms of both shared symbiont lineages and/or functions, forming a deep-sea to terrestrial continuum that also crosses domain frontiers (covering crustaceans, insects, and mammals, at least). The closest relative, *M. schaedleri* has been shown to be involved in host defense in mice, colonizing the mucus that protects gut epithelial cells from bacteria and intestinal content. It outcompetes *Salmonella enterica* serovar Typhimurium colitis for anaerobic respiration limiting its virulence factor expression [76]. Similarly, in *R. exoculata*, the peritrophic membrane prevents direct contact of food and bacteria with epithelial cells. The *Deferribacteres* symbiont might help the *Rimicaris* host preventing pathogen infection. In addition, communication and regulation among symbiont lineages (e.g., through quorum sensing with genes like *luxS* and *luxR*) has been shown in the cephalothorax and digestive *Campylobacteria* epibionts [77]. Although we did not focus on the low abundance *Campylobacteria* in the *Rimicaris* digestive tract in this study, we recovered the *luxS* gene in two of the three *Campylobacteria* MAGs (F_SnakePit_MAG_00005 and M_Rainbow_MAG_00002).

According to our MAG reconstructions and results, we propose new candidate names for these lineages.

##### Hepatoplasmataceae

We propose a new genus and a new species for the three MAGs F_Rainbow_MAG_00001, F_Snakepit_MAG_00004, and F_TAG_MAG_00002 (sharing gANI values of 95.7 to 98.3%, Supplementary Table S5, Fig. 3A): *Candidatus* Foregutplasma rimicarensis, named as such because of their high relative abundance in the *Rimicaris* foregut. Similarly, we propose a new species name for the two MAGs F_TAG_MAG_00001 and F_Snake pit_MAG_00002 (sharing 97.6 to 98.2% gANI): *Candidatus* Bg2_rimicarensis.

##### Deferribacteres

Because of the very low alignment coverage between *M. schaedleri* and our *Deferribacteres* MAGs, due to the reference genomes being too divergent (0.41 to 1.02%, see “ANI alignment coverage Deferribacteres” in Supplementary Table S6, Fig. 3B), we propose here a new *candidatus* fam. nov., gen. nov., and sp. nov. for these lineages: new family *Candidatus* Microvillispirillaceae, named in this way because they are found between microvilli in *Rimicaris* sp., appearing as long spiral-shaped bacteria. The new genus *Candidatus* Rimicarispirillum is named according to the host name and spiral-shaped bacteria. We named M_Rainbow_MAG_00001 and M_TAG_MAG_00002 (sharing ca. 94.8% gANI) *Candidatus* Rimicarispirillum atlantis and M_SnakePit_MAG_00002 and M_TAG_MAG_00004 (sharing ca. 97.6% gANI) *Candidatus* Rimicarispirillum nautilei.

## Conclusions

The discovery of a dual endosymbiosis in the digestive tract of *Rimicaris exoculata* with key nutrition and immune functional marker genes suggests the holobiont is much more complex than previously thought and may explain its colonization success at contrasting sites along the MAR. The cephalothorax and digestive tract microbiomes associated with *Rimicaris* probably communicate and supplement each other when conditions change (in the environment or during different life stages and molt events). Metagenomic plasticity within the framework of these complex symbioses offers the potential for the microbiota to change its composition (addition/loss of microbial members and/or their relative abundance, or symbiont shuffling). It may also allow the holobiont to modulate its gene-expression pattern in response to the host’s physiological changes/life stages and/or to variations in geochemical constraints. Further studies will make it possible to disentangle the functional contributions of these lineages and the host to the overall functioning of the holobiont. Notably, the host genome will reveal its capabilities and interaction pathways with its microbiomes. In addition, metatranscriptomic functional approaches using newly designed in situ sampling tools will be necessary to complement these genomic analyses. Overall, the knowledge gained about this holobiont makes it a keystone species model to establish ecological profiles, which should help with the monitoring of MAR deep-sea ecosystems and to propose preservation strategies in the context of potential anthropogenic impacts on the deep sea.

## Supplementary Information

**Additional file 1: Supplementary Figure 1.** PhyloFlash heatmap of taxonomic assignments (rows, with prokaryotes in blue and eukaryotes in red) for small-subunit rRNA reads in the six individual foregut and midgut metagenomes (columns). The intensity of colors indicates the percentage of reads that mapped to a given taxon. Metagenomes are clustered by their similarity in terms of taxonomic profile and taxa are clustered by their co-occurrence across metagenomes. The Euclidean distance and the Ward's minimum variance method were used for clustering.

**Additional file 2: Supplementary Figure 2.** KEGG Decoder heatmap showing the completeness of the metabolic pathways of the MAGs based on gene presence or absence. The top dendrogram represents the similarity between MAGs based on their metabolic pathways using Euclidean distance and complete linkage clustering. Taxonomic affiliations at the class and phylum levels and MAG completions are indicated at the top of the heatmap.

**Additional file 3: Supplementary Figure 3.** Number of CAZYmes families observed for the different MAG families.

**Additional file 4: Supplementary Table 1.** Description of metagenomes and assemblies. Number of metagenomic short reads sequenced and mapped to the different assemblies and MAGs.

**Additional file 5: Supplementary Table 2.** Description of MAGs. Anvi’o statistics, mean coverage of MAGs and taxonomic assignments obtained with GTDB-Tk.

**Additional file 6: Supplementary Table 3.** MAG single-copy core genes for domain bacteria for each of the 20 studied MAGs.

**Additional file 7: Supplementary Table 4.** List and copy numbers of the genes featured in Figure 4.

**Additional file 8: Supplementary Table 5.** CRISPRs with evidence level 4 and their *cas* genes found in the MAG contigs.

**Additional file 9: Supplementary Table 6.** gANI percent identity and percent alignment coverage between *Deferribacteres* and *Hepatoplasmataceae* MAGs and their most closely related GTDB genomes.

## Data Availability

All data generated or analyzed during this study are included in this published article (and its supplementary information files).
