# Supplementary figures and images for: A novel and dual digestive symbiosis scales up the nutrition and immune system of the holobiont Rimicaris exoculata
Source: Microbiome. 2022 Nov 5;10:189. doi: 10.1186/s40168-022-01380-2 (PMC9636832; doi:10.1186/s40168-022-01380-2)

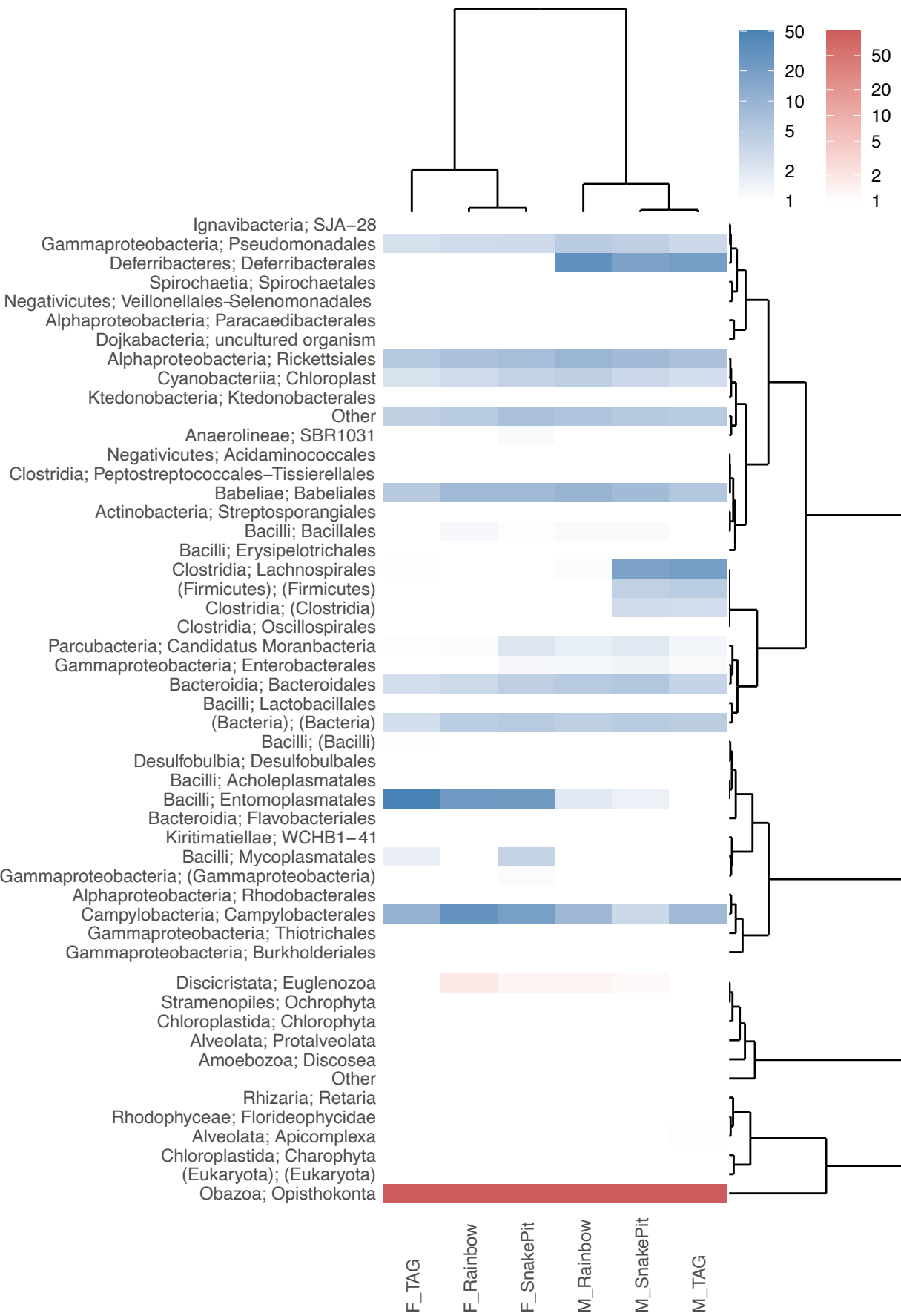

Supplement: Supplementary file 2 — Additional file 1: Supplementary Figure 1. PhyloFlash heatmap of taxonomic assignments (rows, with prokaryotes in blue and eukaryotes in red) for small-subunit rRNA reads in the six individual foregut and midgut metagenomes (columns). The intensity of colors indicates the percentage of reads that mapped to a given taxon. Metagenomes are clustered by their similarity in terms of taxonomic profile and taxa are clustered by their co-occurrence across metagenomes. The Euclidean distance and the Ward's minimum variance method were used for clustering. [file 40168_2022_1380_MOESM1_ESM.pdf]

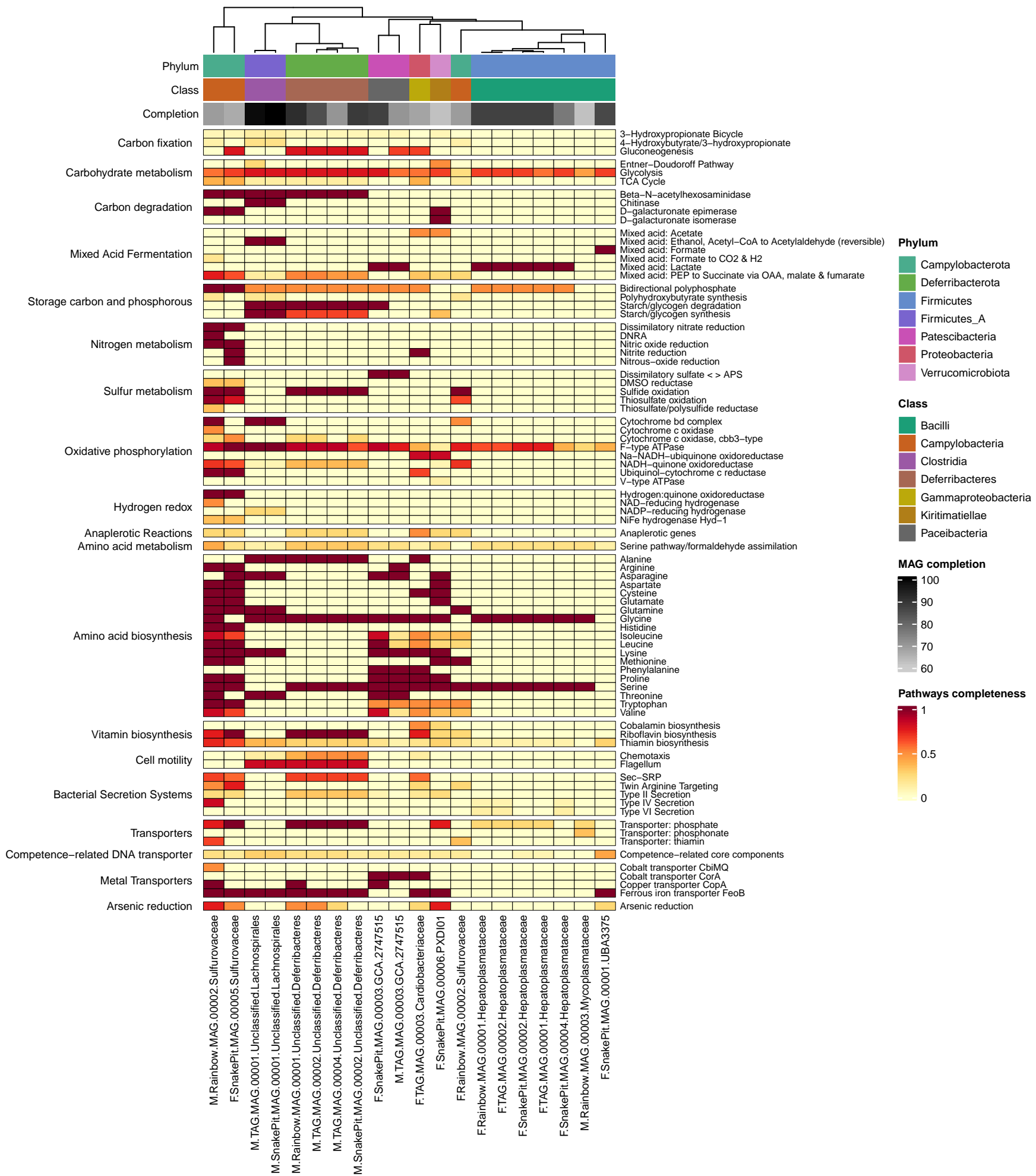

Supplement: Supplementary file 3 — Additional file 2: Supplementary Figure 2. KEGG Decoder heatmap showing the completeness of the metabolic pathways of the MAGs based on gene presence or absence. The top dendrogram represents the similarity between MAGs based on their metabolic pathways using Euclidean distance and complete linkage clustering. Taxonomic affiliations at the class and phylum levels and MAG completions are indicated at the top of the heatmap. [file 40168_2022_1380_MOESM2_ESM.pdf]

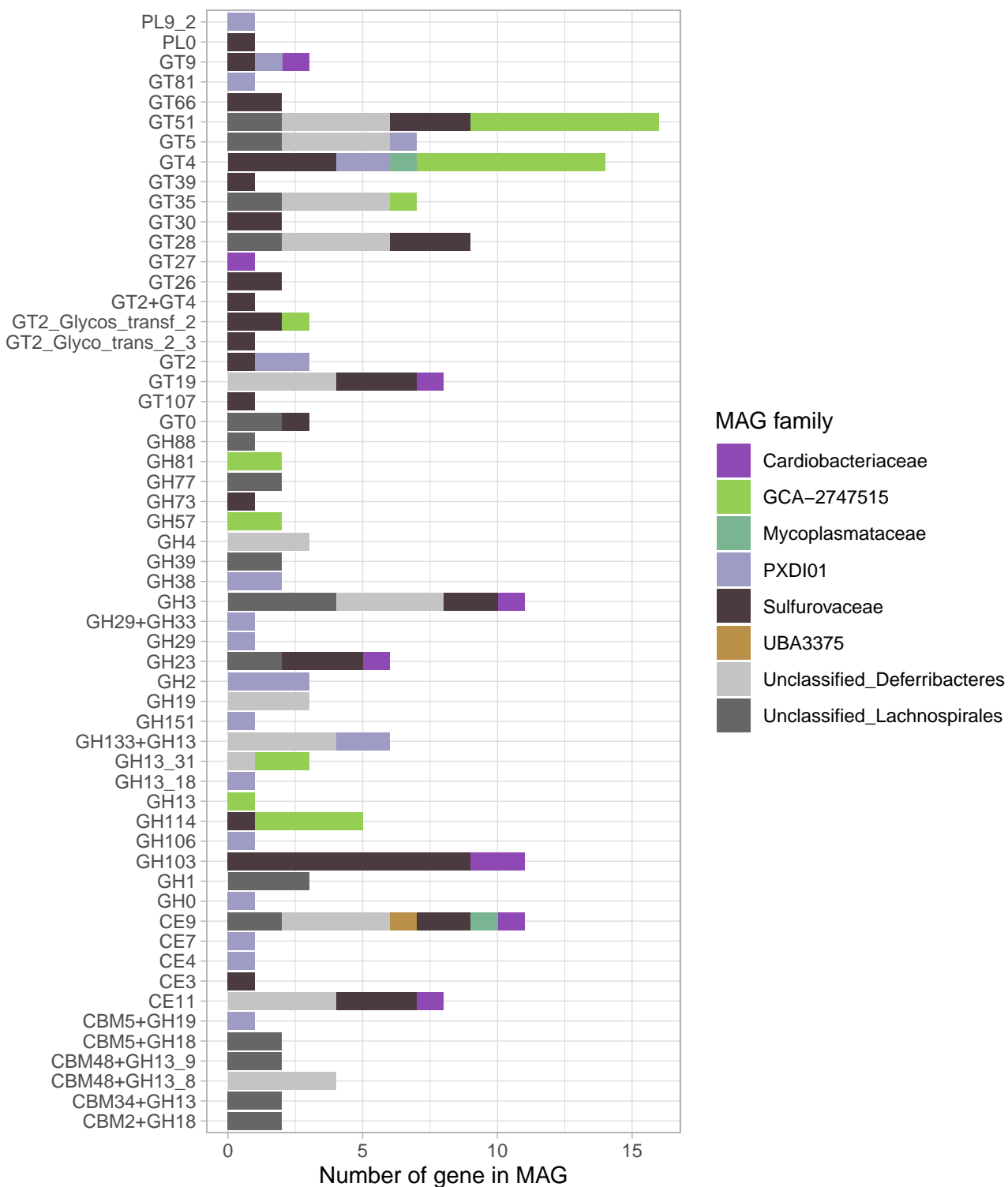

Supplement: Supplementary file 4 — Additional file 3: Supplementary Figure 3. Number of CAZYmes families observed for the different MAG families. [file 40168_2022_1380_MOESM3_ESM.pdf]
